# Trends in US Patients Receiving Care for Eating Disorders and Other Common Behavioral Health Conditions Before and During the COVID-19 Pandemic

**DOI:** 10.1001/jamanetworkopen.2021.34913

**Published:** 2021-11-16

**Authors:** David A. Asch, John Buresh, Kelly C. Allison, Nazmul Islam, Natalie E. Sheils, Jalpa A. Doshi, Rachel M. Werner

**Affiliations:** 1 Division of General Internal Medicine, the University of Pennsylvania, Philadelphia; 2 Leonard Davis Institute of Health Economics, the University of Pennsylvania, Philadelphia; 3 OptumLabs, Minnetonka, Minnesota; 4 Department of Psychiatry, the University of Pennsylvania, Philadelphia; 5 The Corporal Michael J. Crescenz VA Medical Center, Philadelphia, Pennsylvania

## Abstract

This cohort study examines trends in care for eating disorders and other behavioral health conditions before and during the COVID-19 pandemic among commercially insured individuals in the US.

## Introduction

The lay press has reported an increase in eating disorders during the COVID-19 pandemic.^1^ We examined trends in health care for eating disorders from January 1, 2018, to December 31, 2020, alongside other common behavioral health conditions among a large cohort of commercially insured individuals in the US.

## Methods

This cohort study used deidentified data and was deemed exempt by the institutional review board group of UnitedHealth Group. We followed the Strengthening the Reporting of Observational Studies in Epidemiology (STROBE) reporting guidelines.

We counted the unique individuals per 100 000 members per month with outpatient or inpatient care and a primary diagnosis code (*International Statistical Classification of Diseases and Related Health Problems, Tenth Revision *[*ICD-10*]) for eating disorders (*ICD-10*: F50); alcohol use disorders (*ICD-10*: F10); depression, anxiety, and suicidality (*ICD-10*: F33, F34, F40, F41, T14); or opioid use disorders (*ICD-10*: F11). We excluded outpatient claims from members with inpatient claims in the same condition-month and emergency department claims.

## Results

This study included 3 281 366 individuals (2 053 432 females [62.6%]) with a mean (SD) age of 37.7 (16.2) years. Patient characteristics were similar across years, except that the age of patients with eating disorders decreased over time (Table). The number of patients with inpatient care for eating disorders remained approximately 0.3 per 100 000 members per month until May 2020 when it more than doubled to 0.6. This increase was seen across anorexia nervosa, bulimia nervosa, and other and unspecified eating disorders. The median (IQR) length of inpatient stays also increased from 9 (5-17) days and 8 (3-14) days in June to December of 2018 and 2019, respectively, to 12 (5-27) days in the same period in 2020. The number of patients with outpatient care for eating disorders increased from approximately 25 patients per 100 000 per month to 29 patients per 100 000 per month. Similar increases were not seen for the 3 comparison behavioral health conditions (Figure).

**Table.** Patient Characteristics, 2018, 2019, 2020

Condition	Behavioral disorder, No. (%)													
	Eating				Alcohol			Depression/anxiety/suicide				Opioid use		
	2018	2019	2020	2018		2019	2020	2018	2019	2020	2018		2019	2020
No.	19 240	21 496	23 406	73 361		78 647	71 267	1 279 223	1 422 758	1 479 108	54 413		53 077	46 231
Age, y														
0-4	95 (0)	195 (1)	375 (2)	3 (0)		8 (0)	5 (0)	1121 (0)	2152 (0)	2774 (0)	25 (0)		37 (0)	37 (0)
5-17	2034 (11)	2953 (14)	4238 (18)	152 (0)		354 (0)	662 (1)	85 111 (7)	116 661 (8)	137 762 (9)	61 (0)		92 (0)	102 (0)
18-29	7466 (39)	8055 (37)	8425 (36)	16 011 (22)		16 419 (21)	13 533 (19)	301 531 (24)	345 423 (24)	367 229 (25)	9134 (17)		8354 (16)	7278 (16)
30-39	3006 (16)	3448 (16)	3734 (16)	13 653 (19)		15 875 (20)	15 380 (22)	238 093 (19)	281 588 (20)	308 013 (21)	13 250 (24)		13 897 (26)	13 036 (28)
40-49	2918 (15)	3047 (14)	3247 (14)	15 112 (21)		16 980 (22)	16 116 (23)	238 032 (19)	259 590 (18)	266 943 (18)	13 034 (24)		12 995 (24)	11 596 (25)
50-64	3119 (16)	3271 (15)	2990 (13)	22 207 (30)		23 480 (30)	21 251 (30)	299 044 (23)	310 039 (22)	304 889 (21)	14 500 (27)		13 898 (26)	11 415 (25)
65-74	485 (3)	438 (2)	319 (1)	5003 (7)		4456 (6)	3412 (5)	78 595 (6)	71 168 (5)	59 009 (4)	3535 (6)		3002 (6)	2157 (5)
75-84	62 (0)	43 (0)	47 (0)	889 (1)		803 (1)	678 (1)	20 834 (2)	20 596 (1)	19 426 (1)	593 (1)		577 (1)	433 (1)
≥85	55 (0)	46 (0)	31 (0)	331 (0)		272 (0)	230 (0)	16 862 (1)	15 541 (1)	13 063 (1)	281 (1)		225 (0)	177 (0)
Sex														
Females	16 216 (84)	18 105 (84)	19 914 (85)	25 045 (34)		26 550 (34)	24 021 (34)	825 477 (65)	919 823 (65)	975 648 (66)	21 942 (40)		21 091 (40)	18 142 (39)
Male	3024 (16)	3391 (16)	3492 (15)	48 316 (66)		52 097 (66)	47 246 (66)	453 746 (35)	502 935 (35)	503 460 (34)	32 471 (60)		31 986 (60)	28 089 (61)
Region														
Midwest	6065 (32)	7007 (33)	7677 (33)	22 823 (31)		24 491 (31)	22 467 (32)	395 245 (31)	446 733 (31)	459 140 (31)	13 102 (24)		13 871 (26)	12 148 (26)
Northeast	4087 (21)	4627 (22)	4731 (20)	14 049 (19)		14 865 (19)	12 565 (18)	215 730 (17)	240 787 (17)	245 308 (17)	10 069 (19)		9817 (18)	8012 (17)
South	6094 (32)	6672 (31)	7310 (31)	23 279 (32)		25 079 (32)	23 154 (32)	458 728 (36)	507 149 (36)	534 576 (36)	22 659 (42)		20 975 (40)	18 402 (40)
West	2994 (16)	3190 (15)	3688 (16)	13 210 (18)		14 212 (18)	13 081 (18)	209 520 (16)	228 089 (16)	240 084 (16)	8583 (16)		8414 (16)	7669 (17)
Urban location	7584 (39)	8248 (38)	9035 (39)	27 323 (37)		29 225 (37)	25 999 (36)	455 423 (36)	505 252 (36)	529 017 (36)	16 479 (30)		15 753 (30)	13 639 (30)
Zip code level income, $														
≤40 000	461 (2)	502 (2)	485 (2)	1717 (2)		1791 (2)	1609 (2)	39 844 (3)	43 594 (3)	44 009 (3)	2966 (5)		2777 (5)	2485 (5)
40 001 to 60 000	8110 (42)	8918 (41)	9596 (41)	36 026 (49)		38 760 (49)	35 080 (49)	625 958 (49)	695 442 (49)	718 802 (49)	28 986 (53)		28 599 (54)	24 893 (54)
60 001 to 80 000	6426 (33)	7337 (34)	8018 (34)	22 294 (30)		24 213 (31)	22 351 (31)	395 037 (31)	442 211 (31)	466 468 (32)	14 571 (27)		14 313 (27)	12 711 (27)
≥80 001	4243 (22)	4739 (22)	5307 (23)	13 324 (18)		13 883 (18)	12 227 (17)	218 384 (17)	241 511 (17)	249 829 (17)	7890 (15)		7388 (14)	6142 (13)

**Figure.**
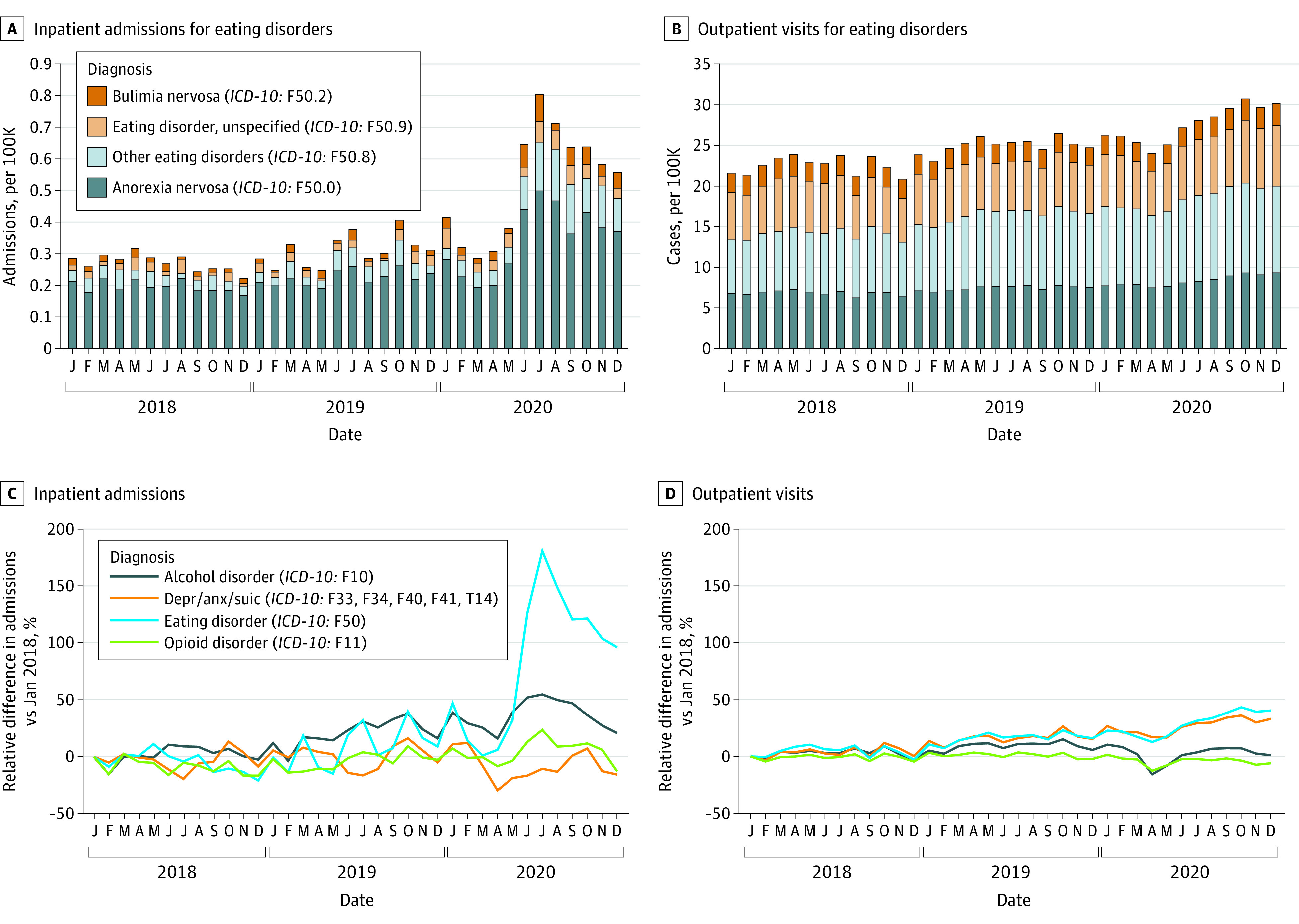
Comparison of Inpatient and Outpatient Visits for Eating Disorders and Their Relative Changes Anx indicates anxiety; depr, depression; *ICD-10*, *International Statistical Classification of Diseases and Related Health Problems, Tenth Revision*; suic, suicide. Claims reflect anorexia nervosa (*ICD-10*: F50.0), bulimia nervosa (*ICD-10*: F50.2), other (*ICD-10*: F50.8), and unspecified eating disorders (*ICD-10*: F50.9).

## Discussion

In this cohort study, we found that inpatient stays for eating disorders rose during the pandemic. Many aspects of the pandemic plausibly intensified eating disorders and their ascertainment. The pandemic may have promoted disordered eating behaviors among susceptible individuals. For example, obesity was frequently cited as a risk factor for COVID severity^2,3^; grocery shopping became more fraught in the early pandemic because of contagion concerns, new rules, and rituals; and many bought large quantities of foods to minimize shopping frequency or fear of shortage.^4,5^ Additionally, exercise may have become a focus of control or a compensatory mechanism for eating.^6^ Furthermore, the closing of schools and colleges may have helped families identify unhealthy eating or recognize its effects, and outpatient care may have been delayed until symptoms required hospitalization. This study was limited because the data was based on the commercial claims of a single insurer.
